# Shoulder Rotational and Dynamic Stability Profiles in Elite and National-Level Tennis Players: A Pilot Study Using an Electromechanical Dynamometer for Measuring Isometric Strength

**DOI:** 10.3390/s25103164

**Published:** 2025-05-17

**Authors:** Álvaro Madroñal-Sotomayor, Luis Manuel Martínez-Aranda, Manuel Ortega-Becerra

**Affiliations:** 1 Department of Sports and Computer Sciences, Faculty of Sports Sciences, Universidad Pablo de Olavide, 41013 Seville, Spain; 2 Science-Based Training Research Group (SEJ-680), Physical Performance and Sports Research Center, Universidad Pablo de Olavide, 41013 Seville, Spain

**Keywords:** injury risk, asymmetry, muscle strength, range of motion, Y-balance, conditioning

## Abstract

Background/objective: Tennis involves repetitive overhead movements, and understanding the relationship between shoulder mobility, dynamic stability, and isometric strength could be crucial for developing targeted training programmes to enhance performance and reduce injury risk. This study aimed to assess shoulder rotational mobility, dynamic stability, and isometric strength profiles in elite and national-level tennis players. Methods: Twenty-four male and female athletes were grouped by competitive level: National-Level Female Group (NFG); National-Level Male Group (NMG); and Elite Male Group (EMG). Shoulder isometric strength was evaluated using an electromechanical dynamometer (Dynasystem), while rotational mobility and dynamic stability were assessed using standardised protocols. Results: Significant anthropometric differences in height, weight, and leg length were identified between NFG and the other groups (*p* < 0.001). NMG showed reduced external rotation compared to NFG and EMG in the dominant shoulder (*p* < 0.05). EMG exhibited significant asymmetries in external rotation between the dominant and non-dominant shoulders, which may be attributed to higher training volumes (*p* < 0.05; ES = 0.994). No significant differences were found in isometric strength across the groups, although NFG showed lower internal rotation strength and ER/IR ratio asymmetry between the dominant and non-dominant shoulder (*p* < 0.05). Dynamic stability scores were consistently low, with asymmetries between the dominant and non-dominant sides in most cases. Conclusions: These findings suggest the need for targeted training to address asymmetries and enhance dynamic stability. Caution is advised when generalising these results due to the limited sample size. Future research should include more participants and explore associations with performance metrics, such as serve speed and playing style.

## 1. Introduction

Tennis is one of the most popular sports worldwide. Unlike most sports, the duration of a match is not predetermined by any time limit. This allows matches to last several hours, requiring athletes to meet physical demands that combine short-intensity efforts with medium-to-high intensity ones. Additionally, the sport involves biomechanical factors related to the execution of various technical gestures in striking and movement, resulting in an injury profile that is not observed in other sports [1].

Acute injuries most frequently occur in the lower extremity, whereas chronic injuries are more common in the upper extremity and trunk [2]. Specifically, shoulder pain is present in 24% of high-level tennis players aged 12 to 20 years, with this prevalence rising to nearly 50% in older athletes [3]. Shoulder joint injuries are primarily attributable to repetitive use or a deficit in glenohumeral internal rotation (GIRD) [4].

Tennis is often introduced at an early age, leading to intensive training that impacts musculoskeletal development. Due to the demands placed on tennis players, which predominantly involve asymmetrical actions, the dominant shoulder is subjected to greater training and competition loads. This results in functional and structural changes that are specific to the dominant side and are not observed on the non-dominant shoulder. A high number of training hours per week (>16 h/week) is associated with an increased risk of injury. Furthermore, a sudden spike in training volume or the number of matches—specifically an increase greater than 60% compared to the previous four weeks—has also been linked to a higher injury risk. This highlights the importance of monitoring the volume, frequency, and intensity of each training and competition session [5]. In this regard, the training volumes reported by adolescent tennis players, ranging from 15 to 20 h per week [6], could increase the injury rate of the dominant shoulder, thus affecting the future performance and career trajectory of athletes [7].

Intrinsic factors associated with an increased risk of shoulder injury include deficits in shoulder range of motion (ROM) and flexibility. Tennis players frequently develop greater glenohumeral external rotation (ER) in the dominant shoulder, whereas internal rotation (IR) in this shoulder is reduced. However, IR is essential in certain actions, such as the serve and groundstrokes, as these involve high demands on the muscles responsible for IR. During the serve, eccentric stretching and pre-tensioning of the anterior shoulder muscles occur [8]. In this movement, shoulder IR is particularly important as it begins before impact and continues into the follow-through phase. The internal rotator muscles must accelerate the upper arm before the external rotators eccentrically contract to decelerate the movement [9]. Combined with the high velocity imparted to the ball and the positioning of the racket by the player, significant loads are placed on the various joints of the tennis player. Therefore, the player must efficiently utilise the kinetic chain with maximum performance in different strokes to minimise the risk of injury [10].

Furthermore, muscle imbalances in the shoulder rotators are commonly observed in athletes engaged in overhead throwing activities. In these athletes, the internal rotators are significantly stronger than the external rotators compared to individuals from the general population or the non-dominant arm. This reduced ER/IR ratio can predict the likelihood of shoulder injuries [11,12,13]. This tendency is also observed in other unilateral or overhead sports, such as handball [14], volleyball [15], or badminton [16], where repetitive movements often contribute to similar patterns of muscular imbalance and associated injury risk.

A range of gadgets and instruments have been employed for evaluating shoulder mobility and power, encompassing manual muscle evaluation, handheld dynamometers, and isokinetic devices. Isokinetic devices are widely regarded as the gold standard for assessing muscle strength due to their capacity to quantify maximum dynamic force across the full spectrum of movement. Electromechanical dynamometry with a functional focus has become a viable alternative for measuring muscle strength in various parts of the body. Studies have characterised it as a trustworthy and accurate method for testing upper and lower limb muscle strength. This technology has been particularly utilised to investigate the isometric strength of shoulder rotators, showcasing high dependability (interrater correlation coefficient > 0.94; coefficient of variation < 10%) [17].

Considering that the lower limbs are primarily responsible for movement and act as the initial link in the kinetic chain during strokes, dynamic imbalances in this region can also increase the risk of injury. Specifically, reduced ankle dorsiflexion can increase the risk of patellar tendon injuries due to decreased load absorption. Additionally, limited ankle dorsiflexion can lead to reduced knee flexion displacement, decreasing quadriceps activation and promoting greater medial deviation and knee valgus [18,19,20]. Therefore, evaluating dorsiflexion ROM is used to identify imbalances or limitations that could represent risk factors and to determine whether the muscle chains are working less efficiently, increasing the likelihood of injuries due to load changes [21]. In this context, functional movement tests are widely used by sports scientists for injury evaluation as they allow for the analysis of mobility, balance, and the detection of asymmetries or functional imbalances. One of the most commonly used tests is the Y-Balance Test (YBT), which assesses mobility in three directions (AN, PM, PL) [22].

Although previous studies have examined shoulder mobility and strength in tennis players, few have simultaneously assessed range of motion, dynamic balance of the lower limbs, and isometric strength of shoulder rotators using a functional electromechanical dynamometer. This study addresses this gap by providing a comprehensive profile of both upper and lower limb function across different performance levels and genders, which could help inform targeted injury prevention strategies. Therefore, the aim of the study was to determine and compare the range of motion and isometric strength in external and internal rotation of the shoulder joint and to assess the dynamic balance of the ankle–knee–hip complex in tennis players of different competitive categories and genders.

## 2. Materials and Methods

### 2.1. Participants

Twenty-four tennis players, both male and female, participated in this study. The players were involved in development and performance programmes at an academy managed by a regional tennis federation. They were assigned to groups according to the regulations of the national tennis federation. Consequently, in this study, participants were grouped by gender and competitive level: national-level female players (NFG, n = 8; age 16.7 ± 0.9 years); national-level male players (NMG, n = 8; age 16.7 ± 1.7 years); and elite-level male players (EMG, n = 8; age 22.1 ± 2.4). Elite players were those who were ranked in the ATP rankings and therefore participated in tournaments organised by this association. In contrast, national players did not require ATP ranking points to participate in state-level tournaments. Elite players trained for an average of 30 h per week, while NH and NM groups trained for an average of 23.8 h per week (SD = 3.5). Regarding experience, elite players had an average of 16.4 years (SD = 3.2), whereas NH and NM groups had 11.5 (SD = 2.5) and 11.0 years (SD = 2.6), respectively.

Regarding inclusion and exclusion criteria, players were eligible for inclusion if they were actively competing at the time of the study, belonged to the training group of the regional federation’s academy, and competed in either the elite or national category. Participants were excluded if they had reported any injury in the six months prior to the study. After being informed about the purpose, testing procedures, and potential risks of the study, written consent was obtained from all participants and from the parents/guardians of minors. No physical limitations, health issues, or musculoskeletal injuries were reported that could have affected the performance of the assessment tests. None of the participants were taking drugs, medication, or dietary supplements that could influence their physical performance. This research was conducted in accordance with the Declaration of Helsinki of the World Medical Association and was approved by the Research Ethics Committee of the University Pablo de Olavide, Seville, Spain (24/8-28).

### 2.2. Experimental Procedures

A quantitative, non-experimental (descriptive), and cross-sectional study was conducted to determine differences in anthropometric characteristics and specific parameters in the shoulder joint and the ankle–knee–hip complex. The aim was to analyse the relationships between these anthropometric and physical fitness parameters within each group of male and female tennis players (female vs. male) of different competitive levels (national vs. elite). Prior to the tests, the participants’ anthropometric measurements were obtained.

All the tests were conducted in a single testing session, during which participants performed an active range of motion tests and isometric strength tests of the shoulder. A dynamic balance test for the lower limbs was also conducted to assess the ankle–knee–hip complex. Measurements were taken for both the dominant and non-dominant segments. To ensure understanding, participants watched demonstration videos of the procedures and performed a familiarisation trial one week before the actual assessment.

The test order was kept constant for all participants. To minimise the potential influence of muscular fatigue on performance, 30-s rest periods were provided between trials within each individual test. Furthermore, the nature of the assessments was not considered physically demanding, as they involved varying levels of participant engagement—passive for range of motion measurements and active for isometric strength and balance tests. Importantly, the active tests targeted different body regions: isometric strength tasks focused on the upper limbs, while balance tests involved the lower limbs, reducing the likelihood of cumulative fatigue. Additionally, a 5-min rest interval was implemented between different tests to ensure adequate recovery. The sessions were conducted under the direct supervision of the researchers and in controlled environmental conditions (20 °C, 60% humidity). Prior to the Maximum Isometric Force (MIF) test, participants completed a standardised warm-up led by the principal investigator. Details of each test are provided below.

#### 2.2.1. Anthropometric Measurements

The following measurements were taken:

(a) Height and body mass were assessed using a fixed scale (Seca, Barcelona, Spain), with an accuracy of 0.001 m and 0.01 kg, respectively.
(b) Dominant leg length was measured from the anterior superior iliac spine to the most distal point of the tibial malleolus using a tape measure (Seca, Barcelona, Spain), with an accuracy of 0.001 m [21].

#### 2.2.2. Range of Motion (ROM)

IR and ER of the shoulders (dominant and non-dominant) were measured using a standard goniometer with a bubble level (Fiasmed, Barcelona, Spain). Participants lay supine on a table with the shoulder abducted to 90°, the elbow flexed at 90°, and the forearm in a neutral rotation. To achieve full joint ROM, the distal half of the humerus was placed off the table.

To measure shoulder IR, the examiner applied downward pressure on the humeral head to stabilise the scapula while participants actively rotated the humerus internally. At the end of the movement, participants were asked to hold the position for several seconds, allowing the examiner to measure the forearm angle by aligning the goniometer with the line between the ulnar styloid process and the olecranon. ER was measured in the same way, with external rotation continuing until the coracoid process began to move [23]. Based on the values obtained for IR and ER, Total Arc of Motion (TAM = IR + ER) and Glenohumeral Internal Rotation Deficit (GIRD = IR non-dominant − IR dominant) were calculated.

#### 2.2.3. Isometric Strength

Isometric strength measurements were taken for both the dominant and non-dominant shoulders using a functional electromechanical dynamometer (Dynasystem Research Model, Symotech S.L., Granada, Spain). The use of this device is supported by previous findings demonstrating its high reliability and validity [17,24]. This device measured the isometric strength of the internal and external rotator muscles of the shoulder [25].

Participants lay supine on a table with the arm vertically positioned, shoulder abducted at 90°, and 0° rotation within the scapular plane. The elbow was flexed at 90°, and the investigator applied downward pressure on the humerus to stabilise it. The angle was initially checked using goniometry and then confirmed by visual inspection.

**Figure 1:**
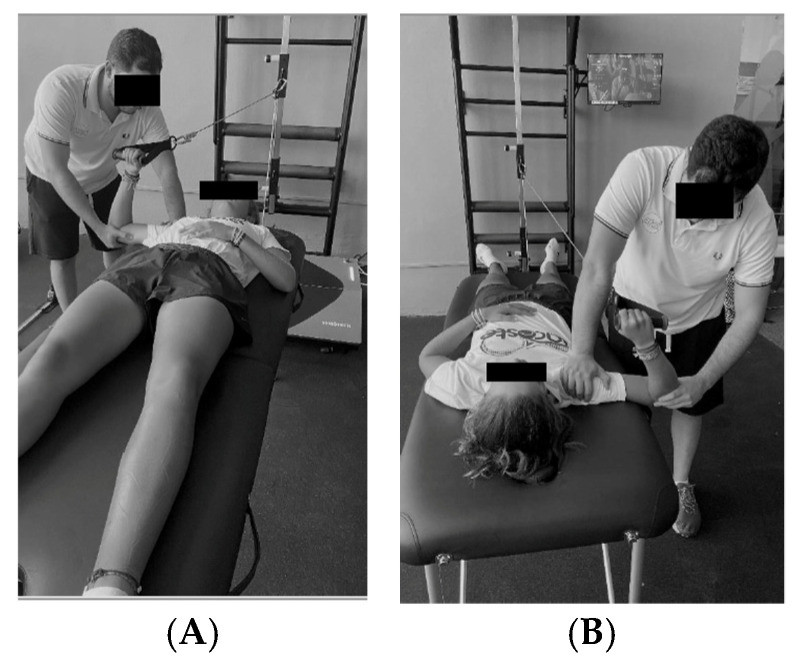
(**A**) Position to perform internal rotation. (**B**) Position to perform external rotation.

To measure internal rotation isometric strength, participants grasped the dynamometer with their head facing the platform and rotated the shoulder internally, as shown in Figure 1A. For external rotation, participants held the dynamometer with their head facing away from the platform and rotated the shoulder externally, as illustrated in Figure 1B [26]. In the isometric strength measurements, the tension in the dynamometer cable provided an immovable resistance, meaning that participants could not generate visible movement. Consequently, for both IR and ER assessments, the action consisted of either pushing or pulling against the cable, which was held via a handle, depending on the movement analysed. For internal rotation, participants grasped the handle and pushed the cable forward across the body, exerting force towards internal rotation. In contrast, for external rotation, they pulled the handle outward, away from the midline, to exert force in the direction of external rotation. The handle and cable remained static throughout, ensuring the assessment was entirely isometric. Participants were instructed to exert maximal force following a standardised sequence directed by the researcher: at the command “ready”, they applied minimal force to preload the cable; at “go”, they exerted maximal force. The peak isometric force was then automatically recorded by the dynamometer software (ver.1.0).

Three isometric contraction tests were conducted for 5 s each, with a 30-s rest interval between the tests. The mean value of the three measurements was calculated, and the data were normalised using the following formula:

- Normalised isometric strength = Maximum force (N, Newtons)/Body mass (kg, kilograms) [6]. (As both variables are expressed in SI units, the resulting value is dimensionless).
- The ER MIF–IR MIF Ratio (%) was computed by dividing the external rotation strength by the internal rotation strength and multiplying by 100 [27]. This ratio was calculated separately for the dominant and non-dominant shoulder to assess potential muscular imbalances.
- ER MIF–IR MIF Ratio (%) = External rotation strength/Internal rotation strength × 100.
- Bilateral asymmetry was defined as a side-to-side difference greater than 10% [6].

#### 2.2.4. Y-Balance Test for Lower Limbs

The Y-Balance Test was conducted to assess the dynamic balance of the ankle–knee–hip complex. Leg length was measured from the anterior superior iliac spine to the most distal point of the tibial malleolus. To simplify the test and avoid potential biases, it was performed barefoot using the Octobalance device (Check your Motion^®^, Albacete, Spain) [21].

Participants were required to maintain balance on one leg while reaching in three directions: anterior (AN); posteromedial (PM); and posterolateral (PL). The subject kept their hands on their waist, did not lift the support foot, maintained balance, and held the starting position for at least one second after the movement. The test was performed on both the dominant and non-dominant leg. Each participant repeated the test three times with each leg, and only the maximum value was recorded [28]. Only correctly executed attempts were considered valid and included in the analysis. An attempt was classified as ‘failed’ and excluded if the participant lifted or shifted the stance foot, lost balance or removed hands from the waist, failed to maintain the final position for at least one second, or touched the ground with the reaching foot before returning to the starting position. Any failed or improperly performed repetitions were excluded to ensure the accuracy and reliability of the measurements. The following calculations were performed:

- Normalised maximal reach distance (%leg length) = (Maximal reach distance [cm]/Leg length [cm]) × 100.
- Composite Score (CS, %) = ((AN + PM + PL [cm])/(LL [cm] × 3)) × 100. A score below 94% indicates a higher risk of lower limb injury.
- Inter-limb asymmetry of the lower limbs (Dom leg − NDom leg value) was calculated, with a difference ≥ 4 cm indicating a higher risk of lower limb injury [28].

### 2.3. Statistical Analysis

Values are presented as mean ± standard deviation (SD). Statistical significance was set at *p* ≤ 0.05. Normality and homoscedasticity were tested using the Shapiro–Wilk and Levene tests, respectively. Statistical differences between groups (NFG vs. NMG vs. EMG) were tested using ANOVA with Bonferroni post-hoc comparisons for normally distributed variables, and the Kruskal–Wallis test with Dunn post-hoc comparisons for non-normally distributed variables. For within-group analyses, the paired Student’s t-test was used for normally distributed variables, while the Wilcoxon test was used for non-normally distributed variables. Effect size (d) was calculated using Cohen’s d, interpreted as follows: small (0.2–0.5); moderate (0.5–0.8); and large (>0.8) [29]). All the statistical analyses were conducted using the free statistical software JASP (Version 0.9.2; University of Amsterdam, Amsterdam, The Netherlands), which is available as open-access software. Please note that this version may not be the most current one at the time of publication.

## 3. Results

### 3.1. Anthropometric Characteristics by Competitive Category

Table 1 shows the anthropometric characteristics of the participants, categorised by competitive level, including height, weight, and leg length. The data indicate that the NFG group shows highly significant differences (*p* < 0.001) in the height variable compared to both EMG and NMG groups. Additionally, significant differences were also observed in the weight and leg length variables when compared to the EMG group (*p* < 0.001 and *p* < 0.05, respectively).

### 3.2. Shoulder Rotation

According to the data shown in Table 2, no significant differences were found in IR (Internal Rotation) between any of the tennis player categories, nor between the dominant and non-dominant shoulder within each group. The reported effect sizes (ESs) were small (ES < 0.50). Regarding ER (External Rotation), no significant differences were observed for the non-dominant shoulder in any of the groups. However, the NMG group showed significant differences (*p* < 0.05) compared to the NFG and EMG groups for the dominant shoulder. In the EMG group, significant differences were found, with a large effect size for ER between the dominant and non-dominant shoulder (*p* < 0.05; ES = 0.994). Moreover, both the NFG and NMG groups showed differences greater than 10% between the dominant and non-dominant shoulder for the ER/IR ratio.

The Total Arc of Motion (TAM) was similar between dominant and non-dominant shoulders across groups, with minimal side-to-side differences. Regarding the Glenohumeral Internal Rotation Deficit (GIRD), the EMG group showed a mean of 6.38° ± 20.34°, the NFG group 7.00° ± 15.51°, and the NMG group 3.13 ± 13.42°. No significant differences were observed between the groups, and the effect size was very low (ES = 0.012), indicating negligible clinical relevance.

### 3.3. Normalised Maximum Isometric Force (NMIF) During External and Internal Rotation

Table 3 presents the results for NMIF (Normalised Maximum Isometric Force). The findings indicate no significant interactions for ER-MIF, IR-MIF, or ER/IR ratio between competitive categories or between the dominant and non-dominant shoulder, except in the NFG group, which showed significant differences (*p* < 0.05) in IR-MIF and ER/IR ratio between the dominant and non-dominant shoulder. Additionally, a significant interaction with the EMG group was also observed (*p* < 0.05).

### 3.4. Y-Balance Test

Table 4 shows the results obtained for the variables related to the Y-Balance Test. No significant interactions were observed between the groups by competitive category. However, it is noteworthy that the reported values for the CS (Composite Score) were significantly below 94%, and the ILA (Inter-limb Asymmetry) was ≥4 cm (considered as indicators of injury risk) in the majority of cases across all three groups of tennis players (see Table 5).

## 4. Discussion

The aim of this study was to assess the anthropometric characteristics and specific parameters in the shoulder joint and the ankle–knee–hip complex in male and female tennis players at different performance levels. The main findings of this study highlighted significant differences between the NFG group compared to the EMG and NMG groups in the evaluated anthropometric characteristics. The NFG group showed lower values for weight, height, and leg length. This was the primary reason for normalising the different variables according to the participants’ weight and leg length, thus avoiding potential biases due to differences between male and female players [27,28,30].

Regarding range of motion (ROM), different patterns were observed for IR and ER. No significant differences were found for IR between the different groups or between the dominant and non-dominant shoulders within each group. However, this was not the case for ER, as the NMG group showed significant differences (*p* < 0.05), with a lower range of external rotation in the dominant shoulder compared to the NFG and EMG groups.

The differences in shoulder range of motion (ROM) between the dominant and non-dominant shoulder were more pronounced in the EMG group, particularly for ER, where significant differences with a large effect size were observed (*p* < 0.05; ES = 0.994). These findings are consistent with previous studies, and it is noteworthy that these differences were even more pronounced in the elite group.

There is ongoing debate about whether asymmetries in shoulder ROM between the dominant and non-dominant sides constitute a risk factor for injury. Some researchers argue that these asymmetrical joint characteristics are part of the sport’s inherent asymmetry and are specific adaptations due to particular strokes, such as the serve and groundstrokes [12]. Additionally, it has been proposed that such adaptations may result from early alterations in shoulder mobility in athletes involved in overhead activities. These changes could be due to adaptive modifications in the proximal humeral anatomy occurring during youth, prior to full epiphyseal plate closure, rather than from degenerative changes in the periarticular structures and soft tissues caused by overuse over time [31].

Conversely, other authors suggest that a difference greater than 5° in the total arc of motion, a deficit in glenohumeral internal rotation (GIRD), or a deficiency in ER (a loss of ER greater than 5° in the dominant shoulder compared to the non-dominant shoulder) could pose a significant injury risk [5,26]. Although this study did not find reductions in IR ROM, as observed in other studies, the values obtained for ER could be potential risk factors for injury in tennis players.

Regarding MIF, no significant differences were observed overall between the different performance levels or between the dominant and non-dominant shoulders. However, the NFG group showed significant differences between the dominant and non-dominant shoulder for IR-MIF and the ER/IR ratio. These findings are consistent with similar studies showing that male players generally exhibit higher strength levels than female players and that greater IR strength is observed compared to ER on the dominant side [6,32]. In relation to the ER/IR strength ratio, Cigercioglu et al. [7] have proposed a threshold below 66% in adult players and below 74% in youth players as potential risk factors for shoulder injuries. In this study, none of the groups exhibited ratios below these values. Therefore, it is considered that there is no significant injury risk related to ER/IR imbalance strength in the evaluated sample.

Similarly to Johansson et al. (2022) [32], when strength values were normalised to body mass, differences in strength levels were either equalised or reduced. Additionally, it was noted that due to the ongoing anthropometric and hormonal changes during adolescence, girls may face greater challenges in maintaining normalised strength levels compared to boys. This may explain the greater differences observed in the NFG group for IR-MIF and ER/IR.

Despite these findings, the absence of significant differences in strength levels overall could be a positive outcome. As these players are in a developmental stage, it underscores the importance of symmetrically developing both the dominant and non-dominant sides to prevent future injuries [33].

Regarding the Y-Balance Test, no significant interactions were observed between performance levels. However, low Composite Score (CS) values and asymmetries between the dominant and non-dominant sides were found. These findings are consistent with previous studies reporting asymmetries that may be indirectly associated with differences in abductor muscle strength, which can affect neuromuscular control and postural stability. For instance, weak hip abductors have been linked to altered lower limb alignment and compensatory movement patterns during dynamic tasks, potentially contributing to balance asymmetries [34]. It is well established that CS values below 94% and Inter-limb Asymmetry (ILA) values ≥ 4 cm may be indicative of mobility dysfunctions and increased risk of lower limb injuries. Nevertheless [28,34], low YBT scores should not be interpreted exclusively as a negative outcome. As suggested by González-Fernández et al. [28], certain sport-specific adaptations—particularly in disciplines with unilateral demands like tennis—may influence postural balance and dynamic stability profiles. Thus, the observed asymmetries and low scores might reflect both a potential injury risk and functional adaptations derived from long-term, unilateral training. This becomes especially relevant considering that all participants were part of similar training environments within the same federation. Therefore, it is essential to further explore individual characteristics, including playing style and limb dominance, to better understand their role in postural control and their implications for injury prevention.

Regarding the Y-Balance Test, no significant interactions were observed between performance levels. However, low Composite Score (CS) values and asymmetries between the dominant and non-dominant sides were found. These findings align with previous studies reporting asymmetries due to differences in abductor muscle strength, highlighting that CS values below 94% and Inter-limb Asymmetry (ILA) values ≥ 4 cm could indicate mobility dysfunctions and an increased injury risk. This finding, in addition to being negative as it suggests a potential risk factor, could also be conflicting given that the participants followed similar training programmes within the same federation/organisation. This raises the question of whether individual characteristics of the tennis players or even their playing style could be contributing to the development of these asymmetries and the increased injury risk. Therefore, it would be necessary to further investigate and analyse these individual factors to better understand their potential impact on injury risk.

However, despite the efforts made by the authors, this study is not without limitations, which should be acknowledged to properly contextualise the findings. Although the sample size (n = 24) was sufficient to detect significant differences in some variables, it was relatively small, which limits the generalizability of the findings. The study was conducted with the available tennis players from a regional tennis federation, which conditioned the size and composition of the sample. Furthermore, given the limited sample size, there is an increased risk of type II errors, meaning that some true differences between groups may not have reached statistical significance. Therefore, it is important to interpret non-significant findings with caution, as larger studies may yield different results. These considerations reinforce the need to regard this investigation as a pilot study aimed at identifying trends and informing future research designs with larger cohorts.

Regarding the inclusion of elite female players, there were no female athletes training at this specific location during the study period. Nevertheless, we fully recognise the importance of incorporating elite female players in future studies to enable a more comprehensive analysis of gender differences, since the scientific literature supports the relevance of sex-based differences in musculoskeletal characteristics and injury risk profiles, especially in youth and elite athletes [6,7,35].

Moreover, due to the cross-sectional nature of the study, causal relationships between asymmetries, balance performance, and injury risk cannot be established. In addition, individual factors such as limb dominance, playing style, and training history were not analysed in detail, although they may influence neuromuscular adaptations. Future research should include longitudinal designs to monitor changes over time and incorporate a broader set of individual variables to better understand the mechanisms underlying postural control and asymmetry. Likewise, integrating functional assessments such as the Y-Balance Test into injury prevention programmes may help coaches and practitioners detect early signs of imbalance and tailor interventions accordingly.

Furthermore, while the study provides relevant functional data, it does not include objective performance metrics (e.g., serve speed, stroke accuracy), which limits the direct interpretation of the findings in terms of on-court performance. This was due to restrictions from coaching and management staff, who did not authorise the evaluation of these variables during training sessions.

## 5. Conclusions

Based on the results obtained in this study, it can be concluded that shoulder joint mobility levels are similar between national-level female players and elite male players, with national-level male players showing the lowest range of shoulder motion. Regarding shoulder strength, the values obtained by the participants are generally comparable, although they are slightly higher in the elite male tennis players compared to the national-level male and female players.

No significant differences were found between the groups in terms of dynamic balance of the ankle–knee–hip complex. However, asymmetries were observed between the dominant and non-dominant sides, as well as low Composite Score (CS) values across the different groups in the study.

Nevertheless, generalising these findings should be approached with caution due to the limited number of players studied. In light of this sample size and the exploratory nature of the analyses, this work should be considered a pilot study. These findings provide preliminary insights that warrant further investigation with larger samples and more detailed performance metrics. Indeed, it would be beneficial to increase the number of participants in each group and to include an elite female players group in future research. Additionally, it would be valuable to investigate the relationships between the variables studied in this work and other factors related to tennis performance, such as serve speed, jumping and sprinting ability, and even playing style.

## 6. Practical Applications

It would be essential to evaluate athletes individually in order to identify their specific risk factors and tailor preventive strategies accordingly. However, some general recommendations for coaches and practitioners can be outlined based on the existing literature [1]. These include: implementing mobility exercises to address any restrictions, particularly in the shoulder, thoracic spine, and hips; incorporating rotator cuff exercises to improve strength, endurance, and muscular balance; strengthening the muscles responsible for decelerating the upper limb during dynamic actions; enhancing strength in the lower limbs and trunk to support force production and improve core stabilisation; and monitoring and managing training load to avoid overuse and optimise recovery.

## Figures and Tables

**Table 1:** Anthropometric variables by groups (Mean ± SD).

	NFG	NMG	EMG
Weight (kg)	62.62 ± 3.02 ^§§§^	70.25 ± 9.28	77.37 ± 4.40
Height (cm)	167.12 ± 4.94 ^§§§†††^	181.25 ± 3.01	181.5 ± 3.46
Leg length (cm)	91.37 ± 3.02 ^§^	94.25 ± 3.58	97.00 ± 3.07

NFG: National Category Female Group; NMG: National Category Male Group; EMG: Elite Category Male Group; ^§§§^ denotes statistically significant differences with EMG (*p* < 0.001); ^§^ denotes statistically significant differences with EMG (*p* < 0.05); ^†††^ denotes statistically significant differences with NMG (*p* < 0.001).

**Table 2:** Shoulder external, internal rotation values, and TAM (dominant and non-dominant) by category (Mean ± SD).

	NFG			NMG			EMG		
	DS	NDS	ES	DS	NDS	ES	DS	NDS	ES
ER (°)	91.62 ± 6.52	87.12 ± 3.52	0.607	85.62 ± 6.76 ^§†^	84.87 ± 6.39	−0.286	96.37 ± 11.75	87.50 ± 9.18 *	0.944
IR (°)	80.00 ± 5.42	83.12 ± 15.02	−0.233	70.62 ± 15.03	77.62 ± 6.76	−0.451	80.87 ± 18.45	87.25 ± 11.58	−0.313
TAM (°)	171.6 ± 6.7	170.3 ± 13.8	0.092	156.3 ± 15.3	162.5 ± 7.8	−0.347	177.3 ± 29.0	174.8 ± 15.7	0.087

NFG: National Category Female Group; NMG: National Category Male Group; EMG: Elite Category Male Group; DS: Dominant shoulder; NDS: Non-dominant shoulder; ES: Effect Size *(d)*; ER: External Rotation; IR: Internal Rotation; TAM: Total Arc Mobility. ^§^ denotes statistically significant differences with EMG (*p* < 0.05); ^†^ denotes statistically significant differences with NFG (*p* < 0.05); * *p* < 0.05 intragroup.

**Table 3:** Normalised Maximum Isometric Force during external and internal rotation and ratio between external and internal (Mean ± SD).

	NFG			NMG			EMG		
N/kg	DS	NDS	ES	DS	NDS	ES	DS	NDS	ES
ER-MIF (N/Kg)	1.22 ± 2.41	1.22 ± 2.65	0.017	1.39 ± 3.93	1.54 ± 4.43	−0.529	1.51 ± 3.75	1.58 ± 4.14	−0.207
IR-MIF (N/Kg)	1.61 ± 2.28	1.31 ± 1.67 *^§^	1.513	1.66 ± 3.02	1.58 ± 3.16	0.288	1.78 ± 3.88	1.71 ± 2.25	0.255
ER/IR (%)	75.57 ± 8.07	94.29 ± 21.93 *	−0.667	84.30 ± 20.45	97.00 ± 17.94	−0.606	87.87 ± 25.39	92.69 ± 20.11	−0.167

NFG: National Category Female Group; NMG: National Category Male Group; EMG: Elite Category Male Group; DS: Dominant shoulder; NDS: Non-dominant shoulder; ES: Effect Size *(d)*; ER-MIF: Normalised External Rotation in Maximum Isometric Force (N/Kg); IR-MIF: Normalised Internal Rotation in Maximum Isometric Force (N/Kg); ER/IR: Ratio External and Internal Maximum Isometric Force (%). ^§^ denotes statistically significant differences with EMG (*p* < 0.05). * *p* < 0.05 intragroup.

**Table 4:** Y Balance Test variables (Mean ± SD).

	NFG			NMG			EMG		
	DL	NDL	ES	DL	NDL	ES	DL	NDL	ES
AN	52.84 ± 2.39	53.35 ± 2.74	−0.197	57.31 ± 3.95	57.61 ± 6.95	−0.054	57.99 ± 7.42	57.29 ± 4.60	0.114
PM	70.18 ± 9.76	75.06 ± 10.82	−0.473	76.25 ± 13.07	75.70 ± 8.85	0.050	80.93 ± 7.54	82.17 ± 11.06	−0.131
PL	79.30 ± 5.78	79.82 ± 9.15	−0.067	80.73 ± 13.82	79.00 ± 8.42	0.152	84.67 ± 6.99	83.58 ± 5.81	0.170
CS	67.44 ± 5.63	69.41 ± 6.43	−0.325	71.43 ± 8.96	70.77 ± 5.79	0.088	74.53 ± 5.84	74.34 ± 6.05	0.031

NFG: National Category Female Group; NMG: National Category Male Group; EMG: Elite Category Male Group; AN: Anterior normalised values; PM: Posteromedial normalised values; PL: Posterolateral normalised values; CS: Composite score; DL: Dominant leg; NDL: Non-dominant leg; ES: Effect Size *(d).*

**Table 5:** Y Balance Test (inter-limb asymmetry).

	NFG	NMG	EMG
	ILA (DL-NDL)	ILA (DL-NDL)	ILA (DL-NDL)
AN	3.0 ± 1.69	4.75 ± 2.31	3.62 ± 3.34
PM	8.62 ± 3.46	4.50 ± 3.94	6.44 ± 3.58
PL	4.0 ± 3.21	7.56 ± 6.67	2.50 ± 1.85

NFG: National Category Female Group; NMG: National Category Male Group; EMG: Elite Category Male Group; AN: Anterior normalised values; PM: Posteromedial normalised values; PL: Posterolateral normalised values; DL: Dominant leg; NDL: Non-dominant leg; ILA: Inter-limb asymmetry.

## Data Availability

The data are contained within the article.
